# COVID-19 lockdown and the rate of central precocious puberty

**DOI:** 10.1007/s40618-023-02146-9

**Published:** 2023-08-11

**Authors:** G. Goggi, M. Moro, A. Chilà, L. Fatti, B. Cangiano, S. Federici, E. Galazzi, E. Carbone, D. Soranna, V. Vezzoli, L. Persani, M. Bonomi

**Affiliations:** 1 Department of Endocrine and Metabolic Diseases, Istituto Auxologico Italiano IRCCS, P.le Brescia 20, 20149 Milan, Italy; 2 Department of Medical Biotechnology and Translational Medicine, University of Milan, Milan, Italy; 3 Biostatistic Unit, Istituto Auxologico Italiano IRCCS, Milan, Italy

**Keywords:** Precocious puberty, Covid-19, SARS-Cov2, Lockdown, Thelarche

## Abstract

**Purpose:**

The aim of our study was to compare the incidence of idiopathic central precocious puberty (CPP) in our highly specialized Endocrinological Center before and after the onset of COVID-19 lockdown; we also aimed to identify any potential difference between girls with CPP from the two different time periods.

**Methods:**

We retrospectively analyzed the auxological profile of 49 girls with idiopathic CPP: 30 with pre-lockdown onset and 19 with post-lockdown onset of the disease. We collected patients’ characteristics (medical history, physical examination, baseline and dynamic hormonal assessment, bone age, pelvic ultrasound) and compared them between the two groups.

**Results:**

We registered an almost threefold increase in CPP incidence in the 2020–2021 period compared to the previous six years. In post-lockdown patients we found a trend for an earlier diagnosis in terms of both chronological age (*p* 0.0866) and days between the onset of first pubertal signs and diagnosis (*p* 0.0618). We also found that post-lockdown patients had a significantly lower hypothalamus-pituitary–gonadal axis activation (lower ∆LH% after GnRH test, *p* 0.0497), a significantly lower increase in bone age calculated at RUS with TW3 method (*p* 0.0438) and a significantly reduced ovarian activation in females (lower delta-4-androstenedione levels, p 0.0115). Interestingly, post-lockdown patients were born from mothers with an older age at menarche (*p* 0.0039).

**Conclusions:**

Besides confirming a significant increase in new diagnoses of CPP in the post-lockdown period, our findings among Post-lockdown girls also suggest a less progressive form of CPP and a stronger environmental influence compared to genetic background in determining the timing of pubertal onset.

**Supplementary Information:**

The online version contains supplementary material available at 10.1007/s40618-023-02146-9.

## Introduction

Precocious puberty (PP) is defined as the onset of pubertal development at an age 2–2.5 standard deviations below the average age of normal puberty, which corresponds to 8 years in females and 9 years in males [1, 2]. The onset of normal pubertal development, however, varies significantly according to several factors, such as children’s ethnicity or body weight [3]. Moreover, several studies have shown that nowadays thelarche in girls appears earlier than it did in the last century [2, 4, 5], while historical data collected in the USA and Europe have shown a significant anticipation of the age of menarche, from about 17 years in the early nineteenth century to about 13 years in the mid-twentieth century [6]. This evidence therefore highlights the role of the environment (as in better socioeconomic conditions, endocrine disruptors, etc.…) in influencing the timing of pubertal onset in children.

At the end of 2019, a new coronavirus—named SARS-CoV-2—was identified in Wuhan, a city in the Chinese province of Hubei, and was responsible for a major wave of new cases of interstitial pneumonia that was named COVID-19 [7, 8]. The virus spread rapidly, causing a global pandemic which led to up to 500 million confirmed diagnoses of COVID-19 [8]. Due to the high rate of spreading of COVID-19, several governments were forced—if reluctantly—to impose restrictive policies promoting social isolation and home confinement to contain the continuous increase of pneumonia cases. The Italian government imposed the national lockdown on March 9th, 2020: as of that moment, the population was compelled to stay at home. Among the consequences of this restrictive measure, it is important to point out a drastic change in children’s lifestyle, which lasted several months [9].

Several Italian and international studies have observed that the incidence of new cases of Central Precocious Puberty (CPP) increased significantly during this period of restrictions, especially in female subjects [9–15]; moreover, the rate of pubertal progression in patients already suffering from CPP also increased compared with the rate observed in the previous years [10]. Despite several hypotheses regarding the mechanism responsible for such an increased incidence of CPP cases following the 2020 lockdown have been put forward, the real pathogenic explanation has yet to be uncovered.

In the present retrospective observational study we therefore meant to compare the incidence of idiopathic central precocious puberty in a single Tertiary Endocrinological Center before and after the onset of COVID-19 lockdown; we also aimed to identify potential difference in anamnestic, clinical, biochemical and/or radiological characteristics between affected girls from the two time periods.

## Materials and methods

### Patients cohort

We evaluated children who were referred by their Primary Care Pediatrician for suspected precocious puberty between 2014 and 2021.

The pivotal inclusion criterion of our study was the diagnosis of idiopathic central precocious puberty based on:

- The appearance of the first pubertal signs within the threshold age for suspecting precocious puberty (thelarche before the age of 8 for females and testicular volume increase over 4 ml before the age of 9 for males);
- A biochemical diagnosis of central precocious puberty (CPP), intended as the finding of LH levels of 5 mU/L or greater (either basal or after stimulation with GnRH) detected within the age of 9 for females and 10 for males;
- Patients who had a known and documented cause explaining the etiology of their central precocious puberty (e.g., CNS lesions or genetic variants known to be pathogenic) were excluded from the analysis.

Patients were then divided into two groups: a first group (Post-lockdown Group) including patients whose first signs of CPP appeared as of the beginning of lockdown in March 2020 until July 2021; a second group (Pre-lockdown Group) comprising patients whose first signs of CPP appeared between 2014 and February 2020.

Considering the paucity of male patients in our cohort, boys were finally excluded from the statistical analysis (yet they were included when calculating the different incidence of CPP between the two time periods).

### Investigations

A detailed family, physiological, pharmacological, past and recent medical history was collected for each patient through the medical reports of their first endocrinological examination following the appearance of pubertal signs: the time of onset of the first signs of pubertal development was retrieved in order to derive the age at puberty onset: as a surrogate for such information, the time when parents became aware of the onset of their children’s first pubertal signs was used. Whenever possible, the age of maternal menarche and patients’ weight at birth were also retrieved. In addition, the presence of any previous or ongoing acute or chronic disease and any drug treatment at the time of the first visit was also investigated; we also took into account whether or not patients were started on treatment with GnRH analogues after the diagnosis of CPP was made. Finally, the time (in days) between the onset of the first pubertal signs and the day the diagnosis of CPP was made (as in the day on which LH values—either basal or dynamic—were found suggestive of CPP) was calculated for each patient.

All patients underwent a thorough auxological and physical examination: the stage of pubertal progression according to Tanner score system, weight, height, BMI, and target height were measured. Height was measured to the nearest tenth of a centimeter using a Harpenden Stadiometer; in patients for whom height measurements prior to the first clinical evaluation were also available, growth velocity (expressed as an increase in centimeters of height in one year) was also calculated. Weight was determined to the nearest tenth of a kilogram using an electronic scale. BMI was also obtained. Target height was calculated except in adopted children. Height, BMI, target height, and growth velocity were all adjusted for chronological age by conversion to SDS (Standard Deviation Score): standard deviations (SD) derived from World Health Organization (WHO) curves were used for BMI; different curves were used for height according to the nationality of each patient: for patients whose parents were both Italian, Italian Cacciari’s curves were used, while for patients who had either one or both parents of foreign origins, WHO curves were used. Finally, the difference in SDS between patient's height and their respective target height (when available) was also calculated.

Blood samples for baseline biochemical assessment were taken fasting and within a time window between 7 a.m. and 10 a.m. Basal LH (U/L), FSH (U/L) and 17β-estradiol (pmol/L) were measured in all patients, using ECLIA (ElectroChemiLuminescence ImmunoAssay) methodology (ELECSYS^®^ technology, Roche Diagnostic). Both LH and FSH assays have a quantification range that goes from 0.1 to 200 U/L, with a lower limit of detection of 0.1 U/L. Whenever available, the levels of other hormones were also collected: total testosterone (nmol/L), delta-4-androstenedione (mcg/L), DHEAS (mg/L), 17-OHP (mcg/L), IGF1 (mcg/L), prolactin (ng/ml), ACTH (ng/L), cortisol (mcg/dL), TSH (mU/L), FT4 (pmol/L) and FT3 (pmol/L). Due to the retrospective nature of our study, the levels of such analytes were not available in all patients and they were sometimes measured in different laboratories using different assay methods. Listed in Supplementary Table 1 is the number of patients in whom it was possible to retrieve such values.

All values of LH, FSH and estradiol included in the data analysis dated back to the day GnRH-test was performed; whenever possible, the same was done for the other analytes as well, otherwise these values were retrieved from earlier samplings, as close as possible to the date on which the GnRH test was performed.

Furthermore, considering the different reference ranges used by different laboratories for the assays of IGF-1, DHEAS, and delta-4-androstenedione and the different reference ranges of IGF-1 according to age, for each of the three analytes the ratio between such values and their respective upper reference limit was calculated and included in the data analysis.

A dynamic assessment of the hypothalamic-pituitary–gonadal axis was performed through GnRH testing: after collecting the Informed Consent from either parent, a peripheral venous catheter was placed within the patient’s mid upper arm (fasting) and a bolus of 100 µg gonadorelin (Relefact^®^ 0.1 mg) was administered intravenously. Blood samples for the measurement of LH and FSH were taken every 30 min (0′; 30′; 60′; 90′; 120′). Basal or post-stimulus LH values above 5 U/L were considered indicative of central precocious puberty. In addition, we aimed to define two further surrogate parameters to investigate the degree of central pubertal activation in our patients: ΔLH and ΔFSH, both in absolute terms and as percentages, were calculated as the difference between LH and FSH peaks and their respective basal values; furthermore LH/FSH ratio was calculated as the proportion of these hormones both at baseline and at their peak.

Bone age was assessed through an X-ray of the left hand and wrist and was then calculated using either Tanner-Whitehouse 3 or Greulich and Pyle method. The difference between bone age and chronological age was then derived for each patient, and it was recorded in both absolute terms and as percentages.

Transabdominal pelvic ultrasonography was performed in order to collect the size and characteristics of uterus and ovaries. Since longitudinal diameter was the only one to be measured in every sonographic evaluation of the uterus, it was the only one that was finally included in our data analysis; in addition, whenever they were available, endometrial thickness and ovarian volume were also collected; ovarian volume was calculated using the ellipsoid formula (longitudinal diameter *x* transverse diameter *x* sagittal diameter × 0. 5233).

Neuroradiological study of the hypothalamic-pituitary region was carried out through a high-resolution nuclear magnetic resonance, both under basal conditions and after administration of intravenous paramagnetic contrast agent. The magnetic field strength used was 1.5 T, with a matrix of 250 × 256. Coronal sections were taken as thick as 2 mm, extending from the posterior wall of the frontal sinus to the sellar tubercle (under basal conditions) and from the tubercle to the sellar dorsum (following the administration of the paramagnetic contrast agent).

The genetic analysis aimed at searching for rare allelic variants that could explain the onset of CPP was performed through a Next Generation Sequencing (NGS) technique using MiSeq^®^ Illumina technology. The panel of candidate genes associated with central precocious puberty that were investigated included the following: MKRN3, GnRH1, GnRH2, GnRHR, KISS1, KISS1R, TAC3 and TACR3.

### Statistical analysis

Normally distributed continuous variables are shown as mean and standard deviation (SD), while non-normally distributed variables are shown as median and interquartile range (IQR) (normality was tested by Shapiro–Wilk). Instead, categorical variables are shown as absolute frequency and percentage. Several statistical tests were used to compare such clinical variables between the two groups of interest: specifically, the *T* test for independent data was used for continuous variables (or Wilcoxon’s nonparametric test in case of non-normality), while the Chi-square test (or Fisher’s test) was used for categorical variables. Finally, the trends of the cumulative number of new cases of precocious puberty for the two periods considered (07/11/2012–29/02/2020 and 01/03/2020–15/07/2021) were represented graphically. Statistical significance was defined as a two-sided *p* < 0.05. Statistical analysis was performed using SAS (version 9.4, SAS Institute, Cary, NC, USA).

## Results

**Fig. 1:**
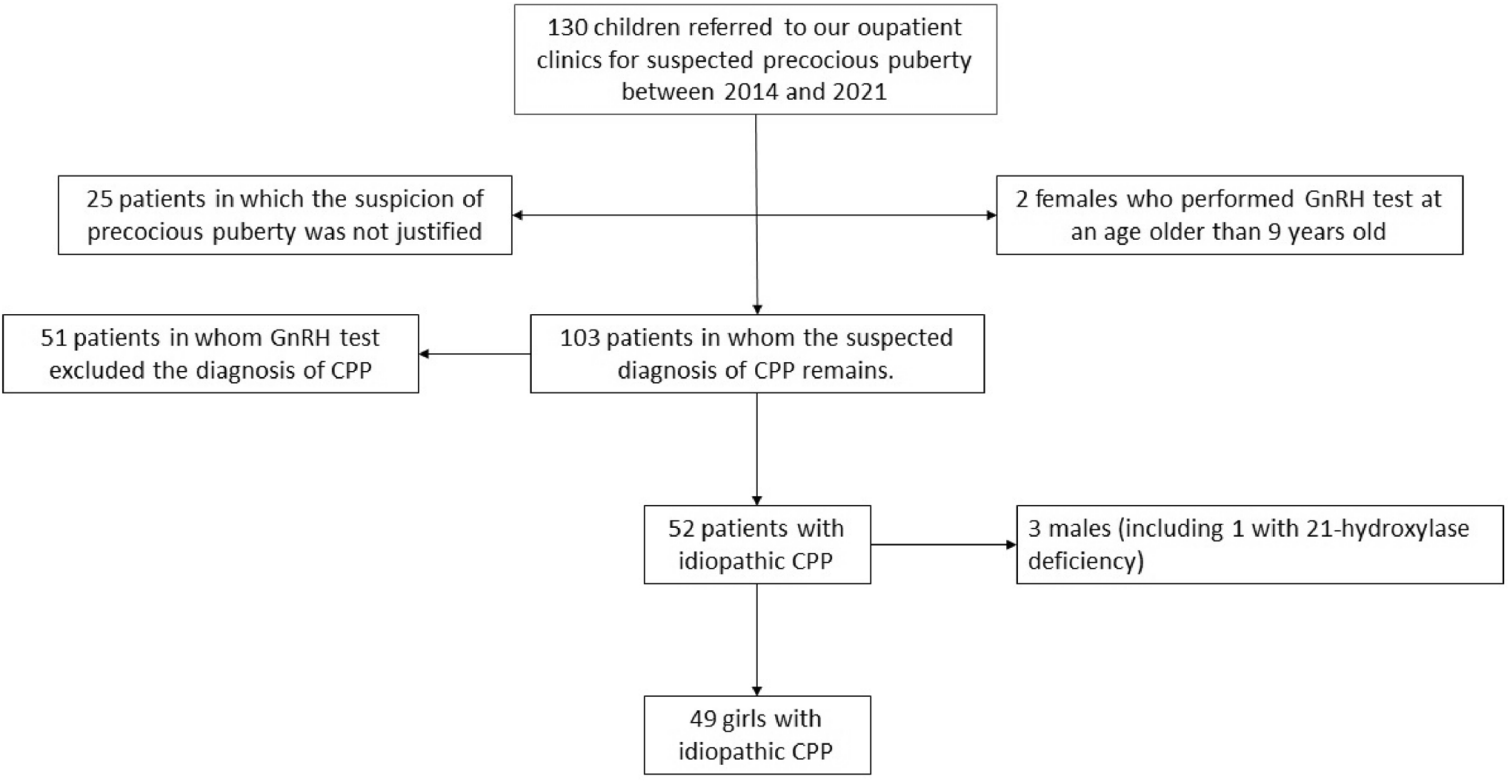
Algorithm for selecting patients to be included in our analysis

According to our inclusion and exclusion criteria, out of 130 children who were referred to our Center for suspected precocious puberty between 2014 and 2021, 81 patients were ruled out for reasons explained in Fig. 1. Thus, a total of 49 girls with CPP were ultimately included in the data analysis and divided into Post-lockdown Group (19 patients, mean age at CPP onset 7.31 years) and Pre-lockdown Group (30 patients, mean age at CPP onset 6.9 years). Post-lockdown Group included 4 foreign-born subjects and 2 girls who were adopted; Pre-lockdown Group, instead, included 3 foreign-born patients, 4 adopted girls, and 1 girl who spent part of her childhood in a country other than Italy (where she had different eating habits).

The comparison between anamnestic, clinical, biochemical, and imaging characteristics of patients in whom precocious puberty started before (Pre-lockdown Group) and after (Post-lockdown Group) the beginning of 2020 lockdown is shown in Supplementary Tables 2–8.

### Incidence of CPP

**Fig. 2:**
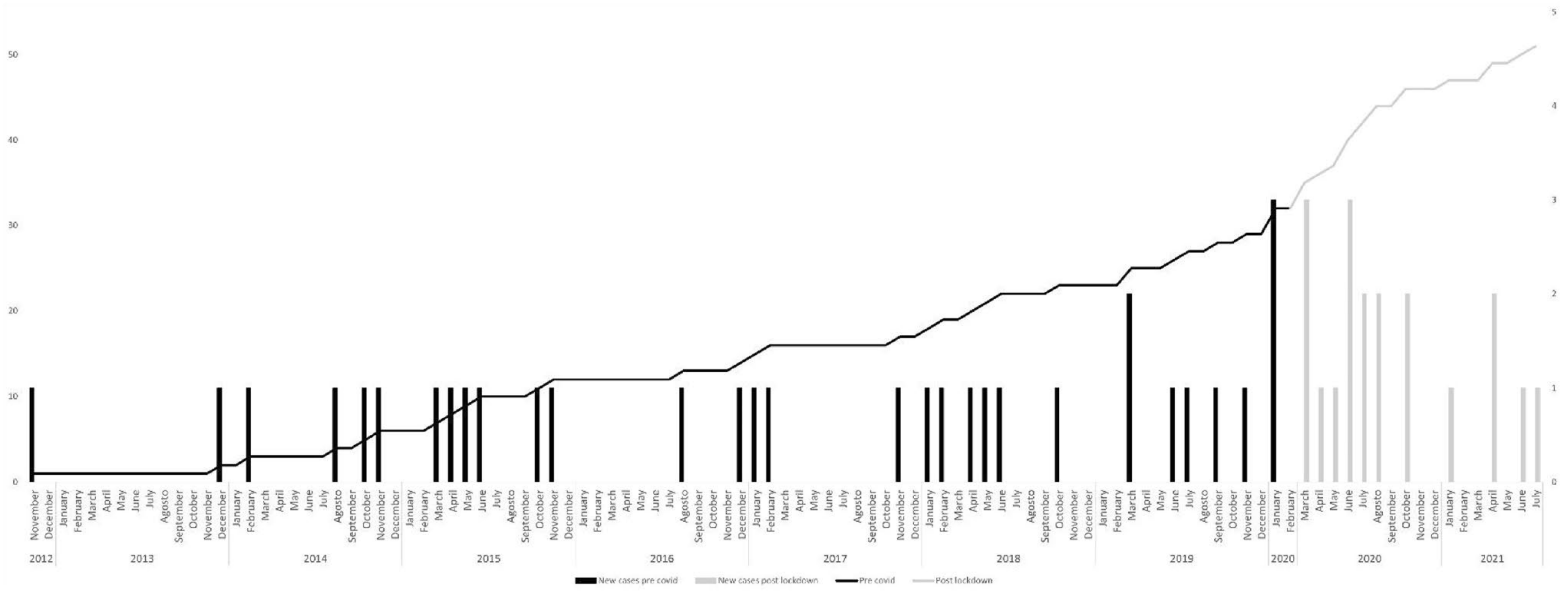
Cumulative and monthly frequencies of new CPP cases in the two time periods considered (pre-lockdown and post-lockdown)

Since March 2020, there has been a significant increase in the number of CPP cases compared to the previous six years (see Fig. 2).

Between the beginning of lockdown and July 2021, our Center recorded the onset of 19 new cases of CPP in 17 months (1.12 cases/month), while in the pre-lockdown period, as of December 2013, it took nearly 57 months (0.33 cases/month) to reach the same number of new cases, which means more than 3 times as much. When considering the whole six-year period prior to the beginning of lockdown, the incidence of central precocious puberty was 0.42 cases/month, which means that as of the imposition of lockdown there was an almost threefold increase in the overall incidence of CPP compared to the pre-lockdown period. Furthermore, at Chi-squared test there was a significant increase in the positivity rate at GnRH testing (*p* = 0.02598) performed in the 2020–2021 biennium compared with those performed within the 2014–2019 period (data not shown).

### Anamnestic data

No statistically significant differences were found between the two groups in terms of age at the onset of first pubertal signs, age at first endocrinological evaluation and weight at birth. Interestingly, the age at menarche of the mothers of Post-lockdown patients was significantly higher compared to that of mothers of Pre-lockdown patients (*p* value 0.0039). Finally, the onset of pubertal signs occurred beyond the age of 7 years in 14 patients in the Post-lockdown Group (74%) versus 19 patients in the Pre-lockdown Group (63%): such difference, however, was not statistically significant (*p* value 0.4516) [see Supplementary Table 2 for more detailed data].

### Clinical and auxological data

The two groups were comparable in terms of weight, BMI, SDS BMI, height, SDS height, delta SDS target height, growth velocity, growth velocity SDS and pubertal stage (P and B stages according to Tanner) [see Supplementary Table 3 for more detailed data].

### Gonadotropins, GnRH test and estradiol

The two groups were comparable in terms of basal LH, basal FSH, peak LH, peak FSH, delta LH and delta FSH in absolute terms, delta FSH in percentage terms, basal LH/FSH ratio, peak LH/FSH ratio and estradiol. However, delta LH value expressed as percentage was significantly lower in Post-lockdown Group than in Pre-lockdown Group (*p* value 0.0497).

Finally, only one patient in Post-lockdown Group (5%) showed a basal LH/FSH ratio > 1 versus no patient in Pre-lockdown Group; as for the LH/FSH ratio at peak, 8 patients in Post-lockdown Group showed a value greater than 1 (42%) versus 16 patients in Pre-lockdown Group (57%): however, for neither of such dichotomous variables a statistical significance was reached between groups (*p* value of 0.3878 and 0.3115 respectively) [see Supplementary Table 4 for more detailed data].

### Other hormones

The two groups were comparable in terms of DHEAS ratio, testosterone, 17OH-progesterone, IGF1 ratio, PRL, ACTH, cortisol, TSH, fT4 and fT3. Interestingly enough, delta-4-androstenedione levels (normalized for the upper limit of the reference range) were found to be significantly lower in Post-lockdown Group than in Pre-lockdown Group (*p* value 0.0115) [see Supplementary Table 5 for more detailed data].

### Bone age

The difference in absolute terms between bone age at RUS calculated with TW3 and chronological age was significantly lower in Post-lockdown Group compared to Pre-lockdown Group (*p* value 0.0438), with a trend towards statistical significance when considering such parameter in percentage terms (*p* value 0.0504); a similar finding was found in Post-lockdown Group also when considering the percentage difference between bone age at CARP and chronological age (*p* value 0.0622). No other statistically significant difference was found between the two groups [see Supplementary Table 6 for more detailed data].

### Pelvic ultrasound

The two groups were comparable in terms of uterine longitudinal diameter, endometrial thickness, and ovarian volume [see Supplementary Table 7 for more detailed data].

### Diagnostic timing, treatment and other investigations

This analysis showed that patients in Post-lockdown Group were diagnosed with CPP earlier than patients in Pre-lockdown Group (in terms of both chronological age at diagnosis and days between the onset of first pubertal signs and diagnosis), with a trend toward statistical significance (*p* value of 0.0866 and 0.0618 respectively); on the other hand, the proportion of patients who were diagnosed with precocious puberty after the age of 7 was comparable between the two groups. Finally, no statistically significant difference was found between the two groups in the proportion of patients who were treated with GnRH analogue: 8 patients from Post-lockdown Group (42%), versus 15 patients from Pre-lockdown Group (50%) (*p* value 0.5895) [see Supplementary Table 8 for more detailed data].

Finally, 24 out of 49 patients underwent MRI of the sellar region (N Post-lockdown Group = 5; N Pre-lockdown Group = 19), but no radiological abnormalities that could justify the onset of CPP were found in any of them (data not shown); similarly, all 9 patients who underwent genetic investigation by NGS (N Post-lockdown Group = 3; N Pre-lockdown Group = 6) resulted negative for pathogenic variants known to be responsible for CPP (data not shown).

## Discussion

Like other Italian and foreign Centers previously did, we also have registered an increase in the incidence of idiopathic CPP: to be specific, an almost threefold increase from March 2020 to July 2021 compared to the pre-lockdown period (from 2014 to February 2020).

At present, there is no certainty about the pathogenic mechanism that was able to trigger such an important increase in the incidence of precocious puberty across different Countries. However, it is speculated that the lockdown itself may have exacerbated the influence of certain known environmental risk factors able to promote the onset of precocious puberty in children, such as weight gain [10, 12, 16], psychological distress [17], an increased exposure to particular endocrine disruptors (such as triclosan, which is contained in soaps and hand sanitizers [15, 18, 19]) and a longer time spent using electronic devices [10, 11].

Those mentioned above are theories about what may have contributed to trigger the significant increase in the incidence of CPP as of the beginning of 2020. It is still not clear which one or which ones of these possible explanations, if any, were actually involved, but most certainly something must have happened during the lockdown that interfered with the normal genetically determined timing of pubertal onset of these children. Consistently with this statement, our study revealed that menarche occurred at a significantly older age in mothers of post-lockdown patients compared to those of pre-lockdown patients (*p* value 0.0039). This certainly constitutes a very interesting finding: indeed, it is well known that the age of pubertal onset is determined by a complex interplay of multiple environmental and genetic factors [20], with genetic factors estimated to be responsible for approximately 50–75% of the variability in the age of onset of the first pubertal signs [21]. A few years ago, a genome-wide association study (GWAS) showed a substantial overlap in genes possibly influencing pubertal timing in both males and females [22], indirectly suggesting that the timing of maternal puberty may in turn be related to the pubertal timing of both sons and daughters [23]. In addition, multiple studies have shown that the age at menarche of mothers is associated with their daughters’ age at menarche as well [23], and a few studies have also assessed a link between maternal age at menarche and the age at each of their either male or female children’s pubertal developmental milestones. For example, a study by Sorensen et al. [23], conducted on more than 15,000 children, showed an association between maternal age at menarche and the age at all pubertal markers in both sons and daughters, a result that appears consistent with the genome-wide association study abovementioned [22]. Similarly, a study by Wohlfahrt-Veje et al. [24] observed that mothers’ age at menarche was associated with both the time of testicular volume increase and pubarche in sons and the time of thelarche in daughters. Based on this evidence, given the higher age at menarche of mothers of post-lockdown patients, we would have expected that in such group thelarche should have appeared later than in Pre-lockdown patients; on the contrary, not only did Post-lockdown patients develop a precocious puberty, but their age at the time of the first pubertal signs did not differ compared to Pre-lockdown Group patients’ (*p* value 0.5449), as if the dissimilarity in maternal age at menarche had no influence at all. This result allows us to hypothesize that the abovementioned postulated environmental risk factors for precocious puberty that were exacerbated by the lockdown may have played a predominant role and a stronger influence than genetics itself in determining the timing of pubertal onset of post-lockdown girls, interfering with what should have been their genetically determined pubertal timing, anticipating its onset. It allows to strengthen the hypothesis that “something” must have happened and impaired the normal onset of puberty, something that goes beyond the mere genetics. Moreover, noting that the age at thelarche in daughters was less strongly associated with parental pubertal timing compared to the age at menarche, the same Wohlfahrt-Veje and colleagues speculated that the age at thelarche might be influenced more by environmental factors rather than genetics [24], which would be in agreement with our hypothesis. One could possibly speculate that the time of paternal puberty might also have influenced the time of pubertal onset of our patients, and that therefore early-developed fathers might have favored a greater precocity of pubertal development in their children, bringing into play an additional variable (about which we have no information in our study). However, again in the study by Wohlfahrt-Veje and colleagues [24] the Authors found that having an early-developed father was not associated with an earlier age at thelarche in daughters, but only with an earlier age at menarche and pubarche. From these results we can therefore hypothesize that paternal influence in the age of thelarche on our female patients was negligible.

Furthermore, in patients who were diagnosed with CPP after the lockdown we found an earlier age at diagnosis and a reduction in the time interval between the appearance of the first pubertal signs and the diagnosis itself compared with patients from previous years: these differences, despite showing only a trend toward statistical significance, are in line with what was observed in the study by de Oliveira Neto et al. [16], in which the time to reach diagnosis was even halved in post-lockdown patients. It is possible that the prolonged period of home isolation gave parents the opportunity to recognize the pubertal changes of their children more promptly; an increased attention to their children’s physical changes was most likely maintained also in the months following the end of lockdown, considering that phenomena such as smart working, e-learning and the persistence of restrictions on social, touristic, sportive and restaurant activities encouraged a further prolongation of the time parents and their children spent together at home. A second explanation, as suggested by de Oliveira Neto et al. [16], could instead be a more rapid progression of pubertal development in post-lockdown CPP patients, which may have made the signs of pubertal development more evident, thus facilitating their discovery by parents and anticipating medical contact. This second hypothesis seems to be in line with what Stagi et al. [10] observed, namely that patients already suffering from CPP experienced an accelerated pubertal progression during and after lockdown. In contrast, in our study both pubic hair and breast development detected at the time of the first clinical evaluation were found to be comparable between the two groups of patients.

Finally, even though the Post-lockdown girls of our cohort presented to medical attention with a bone age advancement by at least 1 year compared to chronological age, and a LH peak after GnRH testing diagnostic for the pubertal range [25], there are several elements that the Authors found suggestive of a less progressive form of CPP compared to Pre-lockdown patients. Firstly, we found that Post-Lockdown girls had a smaller percentage increase in LH (delta LH%) after GnRH testing compared to Pre-lockdown girls. Secondly, Post-lockdown girls had a lower bone age advancement (RUS TW3) compared to Pre-lockdown patients. Lastly, Post-lockdown girls had lower delta4-androstenedione levels compared to Pre-lockdown girls: this was interpreted by the Authors as a lower ovarian activation, since both 17OH-P and DHEA-S levels were normal (therefore ruling out adrenal activation), yet comparable between the two groups (except for one single patient in the Pre-lockdown Group with slightly increased DHEA-S levels). When taken together, all these results lead us to hypothesize that Post-lockdown patients might have undergone a less progressive form of CPP compared to Pre-lockdown patients, although only the long term follow-up of these patients—ideally on a 6–12 months follow-up—will be able to confirm or rule out such hypothesis (taking into account growth-velocity, pubertal development progression, age at menarche, bone age advancement). Hopefully, these observations might be confirmed on multicenter studies.

## Conclusions

In conclusion, our data confirmed an almost threefold increase in new diagnoses of CPP as of the beginning of lockdown compared to the previous six years, suggesting that during the pandemic the influence of genetics in determining the timing of pubertal onset has been scaled back in favor of a stronger environmental influence (the lockdown-effect).

Furthermore, despite a trend towards an earlier diagnosis of CPP was registered during the pandemic, post-lockdown patients seem to be characterized by a less progressive form of pubertal advancement compared to pre-lockdown patients, as suggested by a slightly decreased hypothalamus-pituitary–gonadal axis activation, a less advanced bone age and a significantly reduced ovarian activation (lower delta-4-androstenedione levels). These data would need to be confirmed in a longer observational study focused on auxological follow-up of these children, taking into account hormonal levels, bone age advancement, growth velocity and timing of menarche.

### Supplementary Information

Below is the link to the electronic supplementary material.

Supplementary file1 (DOCX 22 KB)

## Data Availability

Our dataset was published on an online repository (https://doi.org/10.5281/zenodo.7689235).

## References

[1] Harrington J, Palmert MR (2022) Definition, etiology, and evaluation of precocious puberty. UpToDate. https://www-uptodate-com.pros2.lib.unimi.it/contents/definition-etiology-and-evaluation-of-precocious-puberty?search=Definition,%20etiology,%20and%20evaluation%20of%20precocious%20puberty&source=search_result&selectedTitle=1~150&usage_type=default&display_rank=1. Accessed June 2022

[2] Bradley SH, Lawrence N, Steele C, Mohamed Z (2020). Precocious puberty. BMJ.

[3] Biro FM, Chan Y-M (2022) Normal puberty. UpToDate. https://www-uptodate-com.pros2.lib.unimi.it/contents/normal-puberty?search=normal%20puberty&source=search_result&selectedTitle=1~150&usage_type=default&display_rank=1. Accessed June 2022

[4] Sørensen K, Mouritsen A (2012). Recent secular trends in pubertal timing: implications for evaluation and diagnosis of precocious puberty. Horm Res Paediatr.

[5] Teilmann G, Pedersen CB (2005). Prevalence and incidence of precocious pubertal development in Denmark: an epidemiologic study based on national registries. Pediatrics.

[6] Reinehr T, Roth CL (2019). Is there a causal relationship between obesity and puberty?. Lancet Child Adolesc Health.

[7] Wang C, Horby PW (2020). A novel coronavirus outbreak of global health concern. Lancet.

[8] McIntosh K (2022). COVID-19: epidemiology, virology, and prevention. https://www-uptodate-com.pros2.lib.unimi.it/contents/covid-19-epidemiology-virology-and-prevention?search=covid-19%20epidemiology&source=search_result&selectedTitle=1~150&usage_type=default&display_rank=1. Accessed June 2022

[9] Umano GR, Maddaluno I (2022). Central precocious puberty during COVID-19 pandemic and sleep disturbance: an exploratory study. Ital J Pediatr.

[10] Stagi S, de Masi S (2020). Increased incidence of precocious and accelerated puberty in females during and after the Italian lockdown for the coronavirus 2019 (COVID-19) pandemic. Ital J Pediatr.

[11] Chioma L, Bizzarri C (2022). Sedentary lifestyle and precocious puberty in girls during the COVID-19 pandemic: an Italian experience. Endocr Connect.

[12] Chen Y, Chen J (2022). Difference of precocious puberty between before and during the COVID-19 pandemic: a cross-sectional study among shanghai school-aged girls. Front Endocrinol.

[13] Peinkhofer M, Bossini B (2022). Reduction in pediatric growth hormone deficiency and increase in central precocious puberty diagnoses during COVID 19 pandemics. Ital J Pediatr.

[14] Acar S, Özkan B (2022). Increased frequency of idiopathic central precocious puberty in girls during the COVID-19 pandemic: preliminary results of a tertiary center study. J Pediatr Endocrinol Metab.

[15] Mondkar SA, Oza C (2022). Impact of COVID-19 lockdown on idiopathic central precocious puberty - experience from an Indian centre. J Pediatr Endocrinol Metab.

[16] de Oliveira Neto CP, de Azulay RSS (2022). Differences in puberty of girls before and during the COVID-19 Pandemic. Int J Environ Res Public Health.

[17] Xie X, Xue Q (2020). Mental health status among children in home confinement during the coronavirus disease 2019 outbreak in Hubei Province. China. JAMA Pediatr..

[18] Wolff MS, Teitelbaum SL (2015). Environmental phenols and pubertal development in girls. Environ Int.

[19] Harley KG, Berger KP (2019). Association of phthalates, parabens and phenols found in personal care products with pubertal timing in girls and boys. Hum Reprod.

[20] Abreu AP, Kaiser UB (2016). Pubertal development and regulation. Lancet Diabetes Endocrinol.

[21] Zhu J, Kusa TO, Chan Y-M (2018). Genetics of pubertal timing. Curr Opin Pediatr.

[22] Day FR, Bulik-Sullivan B (2015). Shared genetic aetiology of puberty timing between sexes and with health-related outcomes. Nat Commun.

[23] Sørensen S, Brix N (2018). Maternal age at menarche and pubertal development in sons and daughters: a Nationwide Cohort Study. Hum Reprod.

[24] Wohlfahrt-Veje C, Mouritsen A (2016). Pubertal onset in boys and girls is influenced by pubertal timing of both parents. J Clin Endocrinol Metab.

[25] Carel J-C, Léger J (2008). Clinical practice. Precocious puberty. N Engl J Med.

