# Supplementary material for: COVID-19 lockdown and the rate of central precocious puberty
Source: J Endocrinol Invest. 2023 Aug 11;47(2):315–23. doi: 10.1007/s40618-023-02146-9 (PMC10859329; doi:10.1007/s40618-023-02146-9)
Supplement: Supplementary file 1 — Supplementary file1 (DOCX 22 KB) [file 40618_2023_2146_MOESM1_ESM.docx]

**Supplementary Table 1** – Number of patients in whom other hormonal assays were performed

| **Variables** | **Post-lockdown Group** | **Pre-lockdown Group** | **Total** |
| --- | --- | --- | --- |
| Testosterone | 13/19 | 13/30 | 26/49 |
| Delta-4-androstenedione | 17/19 | 18/30 | 35/49 |
| DHEAS | 14/19 | 16/30 | 30/49 |
| 17-OHP | 18/19 | 22/30 | 40/49 |
| IGF1 | 16/19 | 18/30 | 34/49 |
| Prolactin | 7/19 | 10/30 | 17/49 |
| ACTH | 8/19 | 11/30 | 19/49 |
| Cortisol | 8/19 | 11/30 | 19/49 |
| TSH | 19/19 | 28/30 | 47/49 |
| fT4 | 16/19 | 18/30 | 34/49 |
| fT3 | 12/19 | 15/30 | 27/49 |

**Supplementary Table 2** – **Anamnestic data**

| **Variables** | **Post-lockdown Group** | **Pre-lockdown Group** | **p-value** |
| --- | --- | --- | --- |
| Age at onset of first pubertal signs (years) | 7.43 [6.96-7.55] | 7.22 [6.59-7.65] | 0.5449^b^ |
| Age at onset of first pubertal signs > 7 | 14 (74%) | 19 (63%) | 0.4516^c^ |
| Age at first clinical assessment (years) | 7.62 [7.4-7.86] | 7.89 [7.45-8.17] | 0.1660^b^ |
| Age of maternal menarche (years) | 12 [12-13] | 11 [10.5-12] | 0.0039^b^ |
| Weight at birth (g) | 3190.56 (672.86) | 3052 (517.97) | 0.4496^a^ |

Data are represented as means (standard deviation) for normally distributed continuous variables, as medians [interquartile range] for non-normally distributed continuous variables, and as n (%) for categorical variables. a: Student's t-test was used to calculate p-values for normally distributed continuous variables. b: Wilcoxon's test was used to calculate p-values for non-normally distributed continuous variables; c: Chi-square test was used to calculate p-values for dichotomous variables. Statistical significance of differences between groups: p≤0.05.

**Supplementary Table 3** – **Clinical and auxological data**

| **Variables** | **Post-lockdown Group** | **Pre-lockdown Group** | **p-value** |
| --- | --- | --- | --- |
| Weight (kg) | 27.3 [24-36.3] | 29.35 [24.2-34] | 0.7659^b^ |
| BMI (kg/m^2^) | 17.49 (2.54) | 17.62 (1.83) | 0.8462^a^ |
| BMI SDS | 0.81 (1) | 0.91 (0.85) | 0.7173^a^ |
| Height (cm) | 130.25 (8.74) | 129.03 (9.17) | 0.6461^a^ |
| Height SDS (cm) | 0.87 (1.18) | 0.68 (0.99) | 0.5602^a^ |
| Delta SDS Target Height | 1.17 (1.29) | 0.96 (1.1) | 0.57^a^ |
| Growth velocity (cm/y) | 7.63 (1.83) | 7.81 (2.34) | 0.8338^a^ |
| Growth velocity SDS | 2.41 (2.33) | 2.66 (2.85) | 0.8161^a^ |
| Tanner P | 1 [1-2] | 1.5 [1-2] | 0.1469^b^ |
| Tanner B | 2 [2-2] | 2 [2-2] | 0.3810^b^ |

Data are represented as means (standard deviation) for normally distributed continuous variables and as medians [interquartile range] for non-normally distributed continuous variables. a: Student's t-test was used to calculate p-values for normally distributed continuous variables. b: Wilcoxon's test was used to calculate p-values for non-normally distributed continuous variables. Statistical significance of differences between groups: p≤0.05.

.

**Supplementary Table 4** – **Gonadotropins, GnRH test, estradiol and testosterone**

| **Variables** | **Post-lockdown Group** | **Pre-lockdown Group** | **p-value** |
| --- | --- | --- | --- |
| Basal LH (U/L) | 0.6 [0.3-2.2] | 0.6 [0.3-1.5] | 0.6475^b^ |
| Basal FSH (U/L) | 3.8 (1.54) | 3.8 (1.95) | 0.995^a^ |
| Peak LH (U/L) | 9.4 [6.3-26.4] | 14.05 [8.7-26.85] | 0.3186^b^ |
| Peak FSH (U/L) | 12.05 (3.23) | 12.95 (4.43) | 0.4519^a^ |
| Delta LH (U/L) | 8.9 [6-25.9] | 13.2 [8.3-26.4] | 0.2735^b^ |
| Delta FSH (U/L) | 8.25 (3.24) | 9.36 (4.07) | 0.3283^a^ |
| Delta LH (%) | 1566.67 [912.5-1966.67] | 2419.23 [1325-4031.82] | 0.0497^b^ |
| Delta FSH (%) | 214.89 [157.41-395.24] | 265.44 [150.93-533.62] | 0.2931^b^ |
| Basal LH/FSH | 0.19 [0.14-0.47] | 0.24 [0.12-0.37] | 0.7426^b^ |
| Peak LH/FSH | 0.84 [0.62-1.59] | 1.3 [0.84-2.02] | 0.3683^b^ |
| Basal LH/FSH >1 | 1 (5%) | 0 (0%) | 0.3878^d^ |
| Peak LH/FSH >1 | 8 (42%) | 16 (57%) | 0.3115^c^ |
| Estradiol (pmol/L) | 84 [22-124] | 38 [18-66] | 0.1260^b^ |

Data are represented as means (standard deviation) for normally distributed continuous variables, as medians [interquartile range] for non-normally distributed continuous variables, and as n (%) for categorical variables. a: Student's t-test was used to calculate p-values for normally distributed continuous variables. b: Wilcoxon's test was used to calculate p-values for non-normally distributed continuous variables. c: Chi-square test was used to calculate p-values for dichotomous variables. d: Fisher's test was used to calculate p-values for dichotomous variables. Statistical significance of differences between groups: p≤0.05.

**Supplementary Table 5** – **Other hormones**

| **Variables** | **Post-lockdown Group** | **Pre-lockdown Group** | **p-value** |
| --- | --- | --- | --- |
| Delta-4-androstenedione (ratio) | 2.1 [1.85-3.1] | 3 [2.45-4] | 0.0115^b^ |
| DHEAS (ratio) | 0.14 [0.08-0.25] | 0.18 [0.1-0.25] | 0.4927^b^ |
| Testosterone (nmol/L) | 0.2 [0.09-0.25] | 0.09 [0.09-0.29] | 0.9781^b^ |
| 17OH-progesterone (mcg/L) | 0.71 [0.46-0.97] | 0.93 [0.6-1.04] | 0.1779^b^ |
| IGF1 (ratio) | 0.94 (0.38) | 0.8 (0.27) | 0.2237^a^ |
| PRL (ng/L) | 6.4 [5.2-9] | 9 [6.2-10.4] | 0.2222^b^ |
| ACTH (ng/L) | 21.8 [18.4-25.65] | 12.9 [9.1-27.6] | 0.3857^b^ |
| Cortisol (mcg/dl) | 7.98 (2.72) | 8.39 (3.23) | 0.7712^a^ |
| TSH (mU/L) | 2.13 (0.72) | 2.17 (0.63) | 0.8214^a^ |
| fT4 (pmol/L) | 16.95 [14.3-17.65] | 16.05 [15.5-16.7] | 0.3693^b^ |
| fT3 (pmol/L) | 7.68 (0.82) | 7.39 (1.01) | 0.4316^a^ |

Data are represented as means (standard deviation) for normally distributed continuous variables and as medians [interquartile range] for non-normally distributed continuous variables. a: Student's t-test was used to calculate p-values for normally distributed continuous variables. b: Wilcoxon's test was used to calculate p-values for non-normally distributed continuous variables. Statistical significance of differences between groups: p≤0.05; ratio: ratio of the analyte to its upper limit.

**Supplementary Table 6** – **Bone age**

| **Variables** | **Post-lockdown Group** | **Pre-lockdown Group** | **p-value** |
| --- | --- | --- | --- |
| BA according to G&P (y) | 9.43 (1.05) | 8.8 (1.27) | 0.361^a^ |
| Delta BA-CA  G&P (y) | 1.71 (1.33) | 1.47 (0.89) | 0.6852^a^ |
| Delta BA-CA  G&P (%) | 22.91 (19.19) | 20.02 (11.62) | 0.7285^a^ |
| BA according to TW3 RUS (y) | 8.54 (0.92) | 9.2 (1.33) | 0.1062^a^ |
| BA according to TW3 CARP (y) | 8.51 (1.14) | 9.18 (1.24) | 0.1139^a^ |
| Delta BA-CA  TW3 RUS (y) | 0.96 (0.76) | 1.49 (0.73) | 0.0438^a^ |
| Delta BA-CA  TW3 CARP (y) | 1.3 [0.34-1.78] | 1.4 [0.98-1.92] | 0.1833^b^ |
| Delta BA-CA  TW3 RUS (%) | 12.51 (10.04) | 20.06 (11.38) | 0.0504^a^ |
| Delta BA-CA  TW3 CARP (%) | 12.17 (13.03) | 19.79 (10.53) | 0.0622^a^ |

Data are represented as means (standard deviation) for normally distributed continuous variables and as medians [interquartile range] for non-normally distributed continuous variables. a: Student's t-test was used to calculate p-values for normally distributed continuous variables. b: Wilcoxon's test was used to calculate p-values for non-normally distributed continuous variables. Statistical significance of differences between groups: p≤0.05. BA: bone age; G&P: Greulich & Pyle; TW3: Tanner-Whitehouse 3; CA: chronological age; y: years.

**Supplementary Table 7** – **Pelvic ultrasound**

| **Variables** | **Post-lockdown Group** | **Pre-lockdown Group** | **p-value** |
| --- | --- | --- | --- |
| Uterine LL diameter (mm) | 42.49 (7.59) | 40.78 (8.45) | 0.5049^a^ |
| Endometrial thickness (mm) | 1 [0.9-1.2] | 1.2 [0.9-1.8] | 0.2955^b^ |
| Right ovary volume (ml) | 1.92 [1.52-3.15] | 1.9 [1.03-3.54] | 0.5890^b^ |
| Left ovary volume (ml) | 2.24 (0.91) | 2.51 (1.35) | 0.4966^a^ |

Data are represented as means (standard deviation) for normally distributed continuous variables and as medians [interquartile range] for non-normally distributed continuous variables. a: Student's t-test was used to calculate p-values for normally distributed continuous variables. b: Wilcoxon's test was used to calculate p-values for non-normally distributed continuous variables. Statistical significance of differences between groups: p≤0.05. LL: longitudinal; CA: chronological age.

**Supplementary Table 8** – **Diagnostic timing and treatment**

| **Variables** | **Post-lockdown Group** | **Pre-lockdown Group** | **p-value** |
| --- | --- | --- | --- |
| Age at diagnosis (y) | 7.74 [7.42-7.92] | 8.01 [7.54-8.28] | 0.0866^b^ |
| Delta age at diagnosis – age at onset of first pubertal signs (d) | 114 [56-198] | 267.5 [59-403] | 0.0618^b^ |
| Age at diagnosis >7 | 19 (100%) | 28 (87%) | 0.1476^d^ |
| Treatment | 8 (42%) | 15 (50%) | 0.5895^c^ |

Data are represented as medians [interquartile range] for continuous variables not normally distributed and as n (%) for categorical variables. b: Wilcoxon's test was used to calculate p-values for continuous variables not normally distributed. c: Chi-square test was used to calculate p-values for dichotomous variables. d: Fisher's test was used to calculate p-values for dichotomous variables. Statistical significance of differences between groups: p≤0.05. d: days; y: years.
